# Colorectal cancer concurrent gene signature based on coherent patterns between genomic and transcriptional alterations

**DOI:** 10.1186/s12885-022-09627-9

**Published:** 2022-05-30

**Authors:** Ming-Hung Shen, Chi-Jung Huang, Thien-Fiew Ho, Chih-Yi Liu, Ying-Yih Shih, Ching-Shui Huang, Chi-Cheng Huang

**Affiliations:** 1 Department of Surgery, Fu-Jen Catholic University Hospital, No. 69, Guizi Road, Taishan District, New Taipei City, 243 Taiwan; 2 Ph. D Program in Nutrition and Food Science, College of Human Ecology, Fu-Jen Catholic University, No. 510, Zhongzheng Rd., Xinzhuang Dist., New Taipei City, 242062 Taiwan; 3 School of Medicine, College of Medicine, Fu-Jen Catholic University, No. 510, Zhongzheng Rd., Xinzhuang Dist., New Taipei City, 242062 Taiwan; 4 Department of Biochemistry, National Defense Medical Center, No.161, Sec. 6, Minquan E. Rd., Neihu Dist., Taipei City, 11490 Taiwan; 5 Department of Medical Research, Cathay General Hospital, No.280, Sec. 4, Renai Rd., Daan Dist., Taipei City, 106 Taiwan; 6 Division of General Surgery, Cathay General Hospital Sijhih, No. 2, Ln. 59, Jiancheng Rd., Xizhi Dist., New Taipei City, 221 Taiwan; 7 Division of Pathology, Cathay General Hospital Sijhih, No. 2, Ln. 59, Jiancheng Rd., Xizhi Dist., New Taipei City, 221 Taiwan; 8 Division of Hematology and Oncology, Cathay General Hospital Sijhih, No. 2, Ln. 59, Jiancheng Rd., Xizhi Dist., New Taipei City, 221 Taiwan; 9 Department of Surgery, Cathay General Hospital, No.280, Sec. 4, Renai Rd., Daan Dist., Taipei City, 106 Taiwan; 10 School of Medicine, College of Medicine, Taipei Medical University, 250 Wu-Hsing Street, Taipei City, 110 Taiwan; 11 Department of Surgery, Taipei Veterans General Hospital, No.201, Sec. 2, Shipai Rd., Beitou District, Taipei City, 11217 Taiwan; 12 Comprehensive Breast Health Center, Taipei Veterans General Hospital, No.201, Sec. 2, Shipai Rd., Beitou District, Taipei City, Taiwan 11217; 13 Institute of Epidemiology and Preventive Medicine, College of Public Health, National Taiwan University, No.17, Xuzhou Rd., Taipei City, 100 Taiwan

**Keywords:** Colorectal cancer, Concurrent gene signature, Gene expression, Copy number variation, Microarray

## Abstract

**Background:**

The aim of the study was to enhance colorectal cancer prognostication by integrating single nucleotide polymorphism (SNP) and gene expression (GE) microarrays for genomic and transcriptional alteration detection; genes with concurrent gains and losses were used to develop a prognostic signature.

**Methods:**

The discovery dataset comprised 32 Taiwanese colorectal cancer patients, of which 31 were assayed for GE and copy number variations (CNVs) with Illumina Human HT-12 BeadChip v4.0 and Omni 25 BeadChip v1.1. Concurrent gains and losses were declared if coherent manners were observed between GE and SNP arrays. Concurrent genes were also identified in The Cancer Genome Atlas Project (TCGA) as the secondary discovery dataset (*n* = 345).

**Results:**

The “universal” concurrent genes, which were the combination of z-transformed correlation coefficients, contained 4022 genes. Candidate genes were evaluated within each of the 10 public domain microarray datasets, and 1655 (2000 probe sets) were prognostic in at least one study. Consensus across all datasets was used to build a risk predictive model, while distinct relapse-free/overall survival patterns between defined risk groups were observed among four out of five training datasets. The predictive accuracy of recurrence, metastasis, or death was between 61 and 86% (cross-validation area under the receiver operating characteristic (ROC) curve: 0.548-0.833) from five independent validation studies.

**Conclusion:**

The colorectal cancer concurrent gene signature is prognostic in terms of recurrence, metastasis, or mortality among 1746 patients. Genes with coherent patterns between genomic and transcriptional contexts are more likely to provide prognostication for colorectal cancer.

**Supplementary Information:**

The online version contains supplementary material available at 10.1186/s12885-022-09627-9.

## Background

Colorectal cancer (CRC) is the leading cause of human malignancies in Taiwan and ranks third among all cancer deaths [1]. CRC is also a molecularly heterogeneous disease; microarray and reserve transcription-polymerase chain reaction (RT–PCR) experiments have revealed a number of molecular subtypes based on gene expression (GE) profiles, with some displaying associations with disease prognosis or treatment response [2–11]. For instance, Oncotype DX (Genomic Health Inc., Redwood City, CA) was developed as a 12-gene recurrence score. Other signatures included the 18-gene ColoPrint (Agendia Inc., Irvine, CA) and ColoGuide Ex, which is a 13-gene signature using an Affymetrix (Thermo Fisher Scientific, Waltham, MA) exon-based microarray. At the same time, CRC also shows chromosomal instability, with largely unknown clinical significance [12, 13].

The precise etiology of sporadic CRC remains undetermined, as opposed to hereditary familial adenomatous polyposis (FAP) and hereditary nonpolyposis colorectal cancer (HNPCC, ref. [14]). Chromosomal instability might be one of the critical initiatives of sporadic CRC. Cancers can result from progressive accumulation of genetic aberrations with amplified regions containing oncogenes and deleterious regions with tumor suppressor genes. Additionally, cytogenetic analyses have identified oncogenes and tumor suppressors at breakpoints of recurrent chromosomal aberrations [15, 16]. In addition, genomic aberrations could impact GE by complex transcriptional regulation, and genes displaying coherent patterns between the genome and transcriptome are hypothesized to serve as potential biomarkers for prognostication [17, 18].

We used two high-throughput technologies, single nucleotide polymorphism (SNP) and GE microarrays, to conduct an integrated study unraveling critical genes with prognostication in CRC. Although a number of GE signatures have been proposed [2–11], there are unsettled concerns regarding reproducibility and clinical applicability in conjunction with conventional pathological factors [19]. A more sophisticated methodology must be established before molecular signatures can be widely adopted in clinical practice. Herein, we presented a novel GE signature for CRC based on concurrent genes.

## Methods

**Fig. 1:**
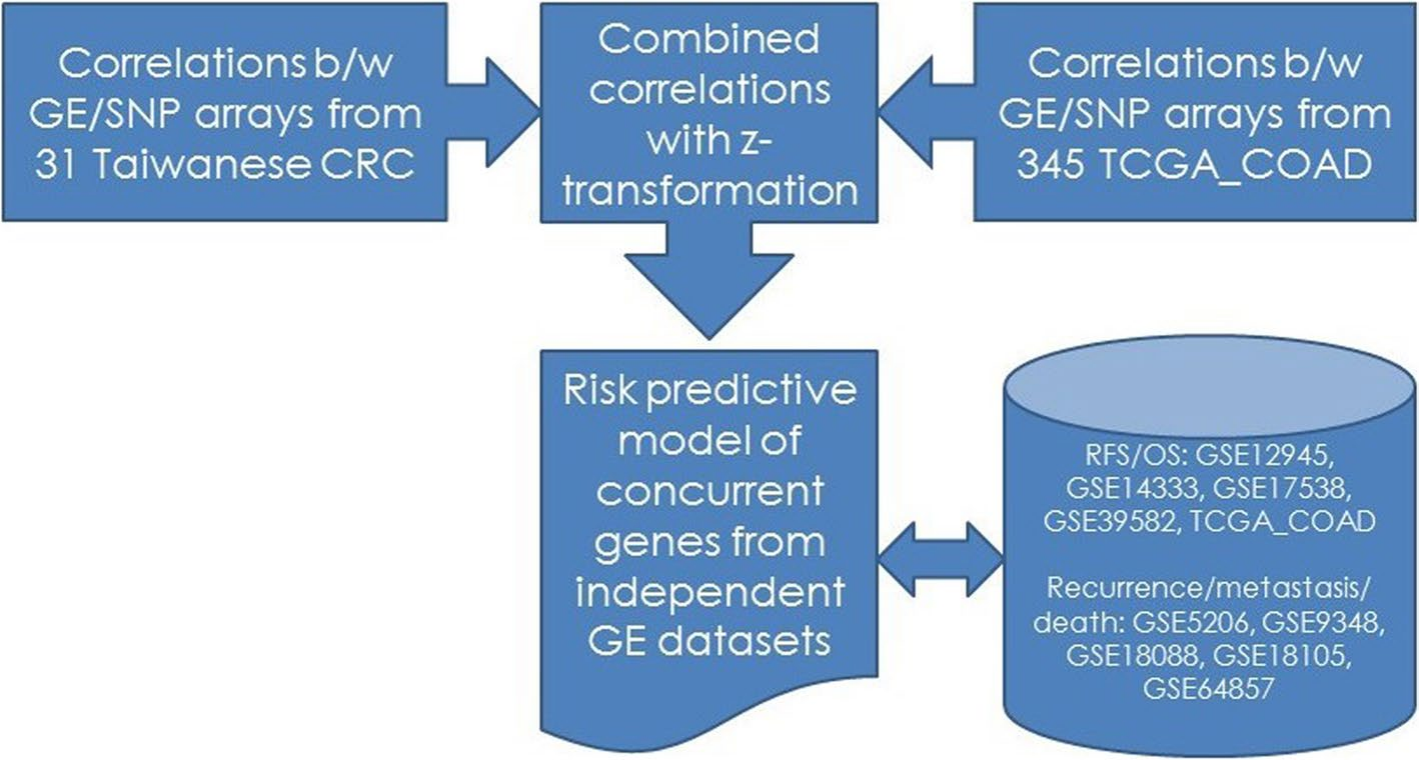
Overview of study design (CRC: colorectal cancer, GE: gene expression, SNP: single nucleotide polymorphism, b/w: between, TCGA-COAD: The Cancer Genome Atlas colon adenocarcinoma, RFS: relapse-free survival, OS: overall survival, GSE: Gene Expression Omnibus series accession number)

The study protocol was reviewed and approved by the institutional review board (IRB) of Cathay General Hospital. Written informed consent was obtained from all the participants after explanation by the investigators (MHS and CCH). An overview of the study design is depicted in Fig. 1.

### CRC samples

CRC samples were prospectively and consecutively collected during surgery. Enrollment criteria included CRC patients who had never received neoadjuvant therapy, were in clinical stages 0 (in situ) to III (no systemic spread) and had no concurrent secondary malignancy. Enrolled patients were managed according to standard guidelines with regular follow-up. All patients with resectable CRC were treated with curative surgeries.

The cancerous tissues were snap frozen and stored in liquid nitrogen below − 80 °C with RNAlater reagent (Qiagen, Germantown, MD) to stabilize RNA. The frozen samples were dissected into slices of 1-2 mm thickness, and more than 90% cancerous content was a prerequisite for microarray experiments.

### Microarray experiments: GE arrays

Total RNA was extracted from frozen specimens using TRIzol reagent (Invitrogen, Carlsbad, CA). Purification of RNA was performed using a RNeasy Mini Kit (Qiagen, Valencia, CA) according to the manufacturer’s instructions. The minimal RNA concentration was set to 100 ng/μl (25 μl) per sample. RNA integration was checked by gel electrophoresis with 2 bands of 18S and 28S indicating satisfactory RNA quality, and an RIN value > 8.0 was pursued to remove heavily degraded samples. Illumina Human HT-12 BeadChip v4.0 (Illumina, San Diego, CA) was used for GE experiments, which provided genome-wide transcriptional coverage of well-characterized genes. Each array on HumanHT-12 v4.0 Expression BeadChip targeted more than 47,000 probes derived from NCBI Reference Sequence (RefSeq) Release 38 (November 7, 2009) and other sources. GE data were normalized using Illumina BeadStudio software Gene Expression Module, and the generated final report files were exported for further analyses, including the AVGSignal spreadsheet.

### Microarray experiments: SNP arrays

DNA was extracted from cancerous tissues using a QIAamp DNA mini kit (Qiagen, Valencia, CA) from the same subject undergoing GE experiments. A minimum of 4 μg DNA was required. A Bioanalyzer 2100 (Agilent, Santa Clara, CA) was used to verify the purity and concentration of cancerous DNA with quality control indicated by OD260/280 > 1.8. Illumina Human Omni 25 BeadChip v1.1 was used for SNP array experiments, which featured approximately 2.5 million markers that captured variants down to a minor allele frequency (MAF) of 2.5% and delivered whole genomic coverage across diverse populations. Structural variations, mainly copy number variants (CNVs), were detected. Illumina HiScan array scanners supported genotyping, CNV, and GE profiling. Projects created with BeadStudio were exported, with three spreadsheets, namely, Genotype, Intensity, and BAlleleFrequency, reported separately.

### CNV detection

CNV detection began with segmentation of normalized data (Intensity spreadsheet from BeadStudio), followed by identification of common (recurrent) gains and losses across multiple SNP arrays. Circular binary segmentation (CBS) was used to identify regions in each chromosome such that copy numbers in each region were equal [20, 21]. The significance level for the test to accept change points was set to 0.01, and the number of permutations was 1000. The Smoothing and MergeLevels algorithm were applied to enhance efficiency [22, 23]. Based on segmented log ratios, the copy number at a particular genomic location was determined using the median absolute deviation (MAD) of log ratios of each array. High-level CNV (amplification and homozygous deletion) was assigned to regions with segmentation mean log ratios > 1 and < − 1 timed the MAD of each corresponding array. The thresholds for low-level CNV (both gains and losses) were 0.5 and − 0.5 MAD, respectively. Pathway enrichment analyses were based on the BioCarta (URL: https://maayanlab.cloud/Harmonizome/dataset/Biocarta+Pathways) database, evaluating the association between a pathway and regions of gain/loss with an empirical *P*-value by 1000 times random sampling.

Regions of recurrent CNV within a cohort of samples were identified using the Genomic Identification of Significant Targets in Cancer (GISTIC, ref. [24]). A null distribution of G scores was generated based on 10,000 resamplings. The significance of CNV at a particular genomic location was determined based on a statistical test obtained from the segmentation log ratios of assayed samples. All bioinformatics analyses of CNV were conducted with the CGH Tools v1.3, part of the BRB-ArrayTools [21]. Results of pathway enrichment and GISTIC analyses were reported for CRC cases assayed for SNP arrays.

### Concurrent gains and losses

Concurrent gains and losses were detected from common genes across SNP and GE microarrays by using HUGO gene symbols as identifiers. The process of mapping between SNP and GE microarray platforms was performed with the SOURCE (URL: https://source-search.princeton.edu/) or Clone/Gene ID Converter (URL: https://cran.r-project.org/web/packages/IDConverter/index.html), depending on which method provided the greatest number of reliable conversions. For probe reduction, multiple probes/probe sets were reduced to one per gene symbol by using the most variable probe/probe set measured by IQR across arrays.

We integrated GE and CNV data to identify genes whose transcriptional abundance was impacted by CNV. A Gene-centric table was outputted by the CGH Tools and detailed the average log-intensity ratio (calculated from all markers within a gene) rather than the discrete CNV status per gene, which was also used to deduce a value corresponding to each gene for each array in the array-covered genomic regions. This value was used to calculate correlations between CNV and GE arrays to distinguish concurrent genes. Concurrent gains and losses were declared if significant changes in a coherent manner were observed for both GE and SNP microarrays (assessed by Spearman correlation coefficients with *P* values < 0.05).

### Concurrent gene signature and classification algorithms

Concurrent genes were identified from Taiwanese CRC and The Cancer Genome Atlas (TCGA) data. The TCGA-COAD (colon adenocarcinoma) Project level 3 dataset of 345 patients was assayed for both GE and CNV profiles using Agilent 4502A (Agilent, Santa Clara, CA) and Affymetrix Genome-wide SNP 6.0 (Thermo Fisher Scientific, Waltham, MA) microarrays. Clinical, CNV, and GE data were downloaded under the synapse ID syn1461155 as the secondary discovery dataset from the URL (URL: https://www.synapse.org/, ref. [25]). Gene mapping was performed as described in Method E, with an additional source of NetAffy (URL: https://www.affymetrix.com/analysis/netaffx_analysis_center_retired.html).

Rather than identifying common genes from both discovery datasets, the “universal” concurrent gene set was derived statistically. Fisher’s z transformation was used to combine correlation estimates from concurrent genes identified from Taiwanese populations and those from the TCGA-COAD dataset with the mathematical formula as follows:

$${\mathrm Z}_r=\tanh^{-1}(r)=\frac12\log\left(\frac{1+\mathrm r}{1-\mathrm r}\right)$$

$$V\left({\mathrm Z}_r\right)=\frac1{n-3}$$

The combined correlations from independent samples were:

$$\overline Z=\frac{\left(n1-3\right)\;z1+\left(n2-3\right)\;z2}{n1+n2-6}$$

$$\overline r=\tanh\left(\overline Z\right)$$

$$V\left(\overline Z\right)=\frac1{\left(n1+n2-6\right)}$$

where *z* is Fisher’s z-transformation, *r* is the sample correlation, *V* is variance, and *n* is the sample size. The universal concurrent gene set was filtered with the predefined threshold of a 10^−3^ α level and was used for downstream prognostic model construction. SAS/STAT software version 15.1 (SAS Institute Inc., Cary, NC) with the CORR procedure was used for the estimation of z-transformed correlation coefficients.

### Microarray datasets

Publicly available microarray datasets were retrieved and fulfilled the purpose of training and validation of the risk predictive model. The primary outcomes were relapse-free or overall survival, and the secondary outcomes were adverse events following curative therapy, such as recurrence, metastasis, or mortality (all were dichotomous outcomes without survival data). Datasets that met the outcome variables were retrieved and are detailed in Supplementary Table 1 (GSE12945, GSE14333, GSE17538, GSE39582, and TCGA_COAD with survival data) and Supplementary Table 2 (GSE5206, GSE9348, GSE18088, GSE18105, and GSE64857 with dichotomous outcomes but without survival data).

### Risk predictive model

A CRC risk predictive model for relapse-free/overall survival was constructed using supervised principal component regression [26]. Concurrent genes were first filtered by the univariate Cox proportional hazards regression, and significant genes within a stringent α level of 0.001 were further used to synthesize the first principal component (supergene), which was subsequently used in risk prediction. A continuous prognostic index score was calculated based on the first principal component for each subject within a dataset, and the high- and low-risk groups were defined by the 50th percentile prognostic index score (noninformative prior). A sensitivity analysis was performed with the prognostic index score cutoff between the predicted high- and low-risk group determined by the lowest censored percentage across all studies (the 75th percentile, Supplementary Table 6).

For dichotomous outcomes such as recurrence, metastasis or death, differentially expressed concurrent genes were identified using the univariate two-sample t test at a 0.001 significance level. A global multivariate permutation test (α level of 10^− 3^) was further used to control false positivity. Multiple methods, including compound covariate predictor, diagonal linear discriminative analysis, 3 nearest neighbors, nearest centroid, and support vector machine (SVM, with default penalty of LIBSVM, ref. [27]), were used to evaluate the prediction accuracy of the CRC risk model (class prediction functions of the BRB- ArrayTools, ref. [21]). For all class prediction methods, leave-one-out cross-validation (LOOCV) was used to calculate the misclassification rate with a permutation *P*-value reported. For each random permutation of class labels, the entire cross-validation procedure was repeated to calculate the cross-validated misclassification rate with the final *P* value determined from the proportion of the random permutations giving the least misclassification rate. A minimum of 1000 permutations was required.

As distinct statistic/bioinformatics tools were adopted with different underlying hypotheses and corresponding scenarios, there was no uniform alpha-level across all these tests. Consequently, default alpha-level of each test from the BRB-ArrayTools was followed. Usually reduced α levels were required for multiple testing (Bonferroni correction). Model training and LOOCV were performed within each study, and consensual genes across all microarray datasets were used to build the CRC risk predictive model. Genes were median-centered first within each dataset to avoid introducing bias from extremely high intensities as well as batch effects.

## Results

### Taiwanese CRC cohort

A total of 88 CRC patients were recruited during the study period between October 2013 and May 2016. There were 51 males and 37 females, with a median age of 63 (range: 33-88) years. There were 81 adenocarcinomas, 2 mucinous adenocarcinomas, and 5 in situ lesions. The anatomical distributions were ascending (21), transverse (9), descending (10), sigmoid (19), rectosigmoid (9), anorectum (18), and overlapping lesions (2). There were 46 low-, 32 intermediate-, and 7 high-grade cases. During the follow-up period of up to 4 years, there were 7 recurrences and 14 metastases.

### CNV of 32 Taiwanese CRC patients

**Fig. 2:**
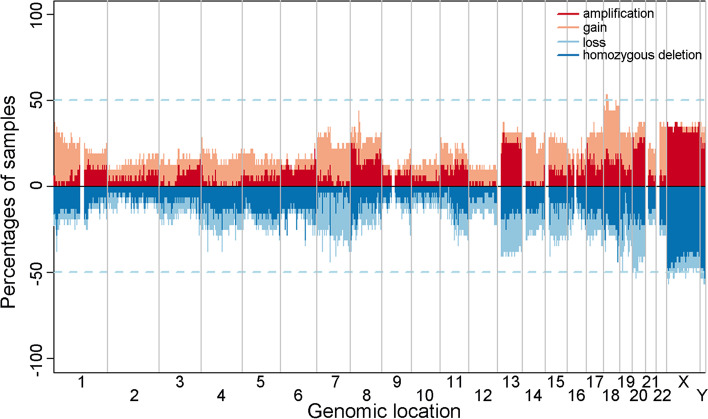
Frequency plot of CNV among 32 Taiwanese CRC patients (CNV: copy number variation, CRC: colorectal cancer)

The number of unique markers delivered by the Illumina SNP array was 2,267,360, and the frequencies of CNV among 32 Taiwanese CRC patients (31 also assayed for GE) are displayed in Fig. 2. Supplementary Fig. 1 summarizes gain and loss calls on the genome detected on each array. Pathway enrichment analysis with BioCarta showed that there was one pathway enriched in genes with gain and two pathways enriched in genes with loss (Supplementary Table 3, ref. [28]). Frequent CNVs (gain regions) identified by GISTIC are detailed in Supplementary Table 4, including 13q12.2-q12.3 (*PDX1*, *ATP5EP2*, *CDX2*, *PRHOXNB*, *FLT3*, *LOC100288730*, *PAN3*, and *FLT1*) and 17q12-q21.2 (*NEUROD2*, *PPP1R1B*, *STARD3*, *TCAP*, *PNMT*, *PGAP3*, *ERBB2*, *C17orf37*, *GRB7*, *IKZF3*, *ZPBP2*, *GSDMB*, *ORMDL3*, and *LOC728129*).

### Consensus of universal concurrent genes

The number of concurrent genes was 1582 (*P* < 0.01) and 2974 (*P* < 0.01) from the Taiwanese (*n* = 31 for CRC patients assayed for both platforms) and TCGA-COAD (*n* = 345) cohorts, respectively, resulting in a common concurrent gene set of 307 genes. The universal concurrent genes, which were synthesized from the combined correlation coefficients with z-transformation, contained 4022 genes at the *P* < 0.001 level. These candidate genes were evaluated within each of the 10 microarray datasets, and 1655 (2000 probe sets) were filtered as being prognostic in at least one study (Table 1). The consensual genes across studies were used to build a CRC risk predictive model with a significance level determined by a nominal univariate test at 0.01 α level. Candidate genes decreased from 1365 to 1 when the number of agreeing datasets increased from 1 to 5 (Table 2). Finally, a subset of 49 consensual concurrent genes were incorporated into the CRC prognostic model, which was determined by cross-validation. A complete list of the 49 constitutional genes is detailed in Supplementary Table 5.

**Table 1:** The number of prognostic concurrent genes (probe sets) within each training dataset

Dataset	Number of prognostic probe-sets	Percentage of prognostic probe-sets
GSE12945	25	1.25%
GSE14333	251	12.55%
GSE17538	53	2.65%
GSE18088	1005	50.25%
GSE18105	48	2.4%
GSE39582	1	0.05%
GSE5206	338	16.9%
GSE64857	68	3.4%
GSE9384	168	8.4%
TCGA_COAD	43	2.15%

**Table 2:** The number of consensual concurrent genes among training datasets

Number of consensual datasets	Number of concurrent genes
5	1
4	4
3	44
2	241
1	1365

### Survival analysis

**Fig. 3:**
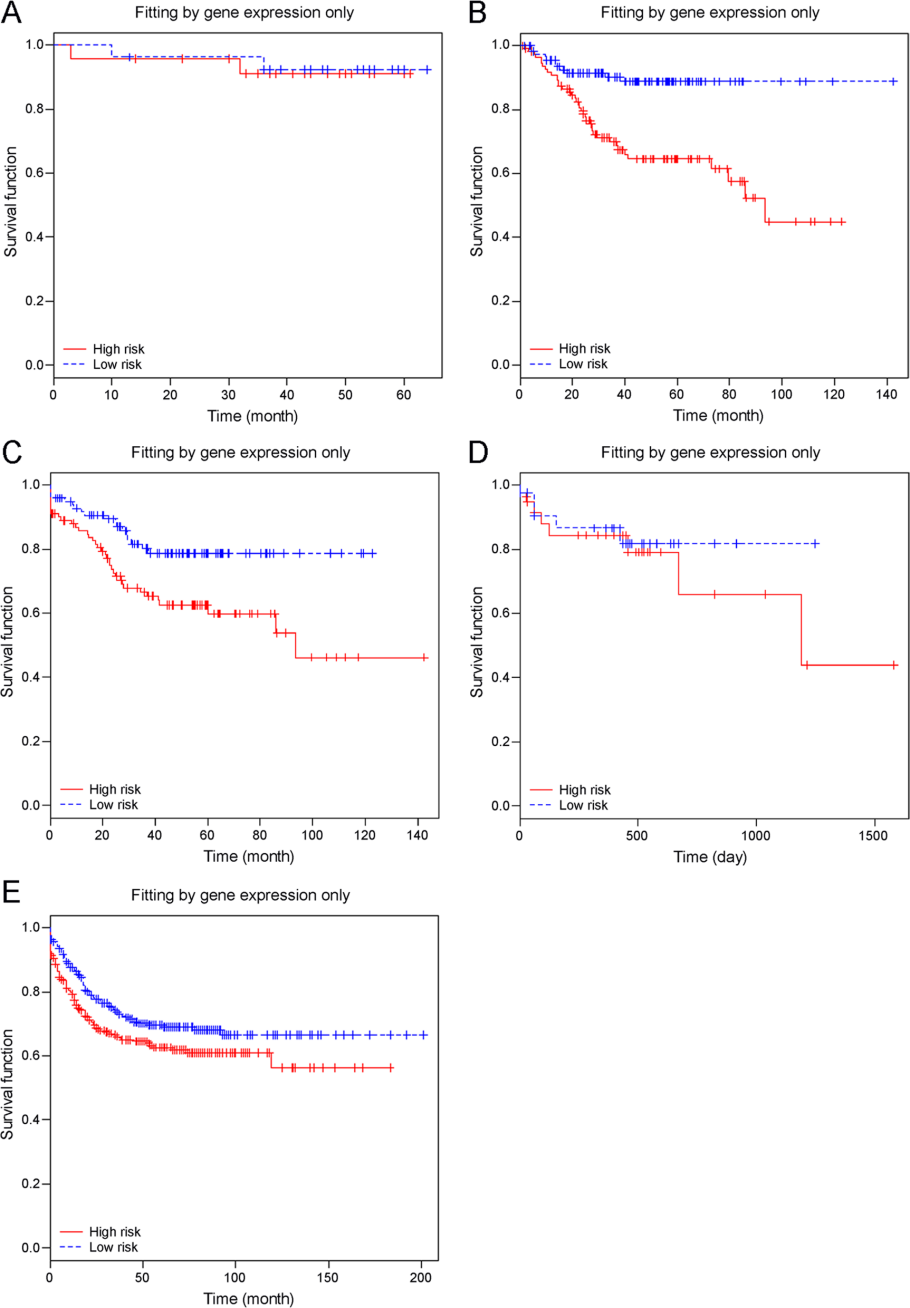
**A** to **E** Relapse-free/overall survival analysis from microarray datasets of GSE12945 (**A** top left), GSE14333 (**B** top right), GSE17538 (**C** middle left), TCGA_COAD (**D** middle right), and GSE39582 (**E** bottom) with leave-one-out cross-validation. The high−/low-risk group was defined by the 50th percentile of the prognostic index score determined by the supervised principal component composed of 49 concurrent genes. All survival times were measured in months, except for 3D, which was measured in days

A supervised principal component encompassing 49 consensual concurrent genes was used for survival analysis, with the threshold of the 50th percentile of the prognostic index score for risk group construction with LOOCV. Figure 3A to E show relapse-free/overall survival patterns from the GSE12945, GSE14333, GSE17538, TCGA_COAD, and GSE39582 datasets. Except for GSE12945 (log-rank test: *P* = 0.82), survival discrepancies were observed in 4 out of 5 studies between defined high- and low-risk groups (log-rank test: *P* < 0.0001, *P* = 0.0059, *P* = 0.049 and *P* = 0.0361, respectively). In order to evaluate the impact of the 50th percentile thresholding, Table 3 summarizes area under the curve (AUC) from time-dependent receiver operating characteristic (ROC) curve, as well as censored/uncensored number from each study. The highest AUC, 0.824, was reported from TCGA_COAD cohort; it should be noticed that it was also this cohort contributed much more samples during the discovery of concurrent genes. A differential bias toward favorable prognostic power for this cohort should be considered.

**Table 3:** Summary of censored case numbers in high- and low-risk groups and area under the curve (AUC) from time-dependent receiver operating characteristic (ROC) curve from survival analysis (gene only model, *RFS* Relapse-free survival, *OS* Overall survival, *CRC* Colorectal cancer, *GEO* Gene expression omnibus)

CRC case number	GEO	Outcome	Predicted high−/low-risk patients	Censored in high-risk group	Censored in low-risk group	Landmark time	AUC
51	GSE12945	RFS	24/27	22(91.7%)	25(92.6%)	49	0.672
226	GSE14333	RFS	113/113	74(65.5%)	102(90.3%)	38.5	0.643
200	GSE17538	RFS	101/99	64(63.4%)	81(81.8%)	39	0.63
174	TCGA_COAD	OS	89/85	79(88.8%)	79(92.9%)	30.5	0.824
557	GSE39582	RFS	279/278	182(65.2%)	198(71.2%)	43	0.519

To further evaluate the impact of different thresholds on risk grouping, a sensitivity analysis was performed with a different cutoff of the 75th percentile. Supplementary Table 6 shows the number of predicted high−/low-risk patients and censored cases within each risk group while survival patterns and AUC are detailed in Supplementary Fig. 2. It deserved noticed that with an informatic prior of one-quarter of the assayed samples with the highest prognostic index score being high-risk, discerning ability of the purposed signature was much compromised, and only two out of the five studies with follow-up data showed a significant log-rank test (Supplementary Table 6). These two studies, GSE14333 and GSE 17538, were with a proportion of censored cases most similar to the pre-selected 75th percentile cutoff and reported an optimistic survival advantage among CRC patients predicted into the low-risk group (Supplementary Fig. 2B and C).

### Predictive accuracy of CRC risk model

Table 4 shows the predictive accuracy of the concurrent gene-based CRC prognostic model with multiple methods predicting adverse events of recurrence, metastasis, or death during the follow-up period. In general, the Bayesian compound covariate model delivered the best predictive accuracy, with a cross-validation AUC reaching 0.833.

**Table 4:** CRC risk predictive accuracy of recurrence, metastasis, or death by the concurrent gene prognostic model

CRC case number	GEO	Outcome	Compound covariate (%)	Diagonal linear discriminant (%)	1-NN (%)	3-NN (%)	Nearest centroid (%)	SVM (%)	Bayesian compound covariate (%)	CV-AUC from Bayesian compound covariate
100	GSE5206	Recurrence	82	83	75	80	80	77	86	0.833
70	GSE9348	Metastasis	70	76	79	81	70	73	85	0.768
53	GSE18088	Relapse	75	75	75	75	77	72	89	0.863
111	GSE18105	Metastasis	57	53	66	60	56	61	N/A	0.548
75	GSE64857	Recurrence	61	60	64	64	63	57	81	0.684

*CRC* Colorectal cancer, *GEO* Gene expression omnibus, *NN* Neural network, *SVM* Support vector machine, *CV-AUC* Cross-validation area under receiver operating characteristic curve, *N/A* Not applicable

## Discussion

CRC is a major gastrointestinal malignancy, while its development and progression involves a complex process with multiple genetic changes. Therefore, deciphering the molecular heterogeneity of CRC will contribute to accurate risk assessment and identify effective therapies. Mainstays of CRC management include early detection by screening (fecal occult blood testing), complete surgical resection of the lesion with regional lymph node dissection, and adjuvant systemic therapy based on the diagnostic stage. Adjuvant therapy, usually with the form of cytotoxic chemotherapy, is mainly determined by the pathological staging system including depth of tumor invasion, regional node involvement and distant metastatic status. Usually stage I CRC is managed with regular surveillance, stage III is deemed with adjuvant therapy; while for stage II disease, there remains a lack of prognostic biomarkers for risk assessment except dMMR (mismatch repair deficiency) status [29].

Beyond anatomical staging, GE patterns of CRC may provide additional prognostication, which is complementary to pathological features. This biological staging will not be achievable without a transcriptome-based molecular profiling. The current study evaluated the prognostic value of concurrent gene sets specific for CRC, and a risk predictive model was proposed. We developed an analytical approach to identify genes with coherent patterns between transcriptome and CNV profiles using matched GE and SNP microarray data to reduce false discoveries in concurrent gene signatures.

CRC is heterogeneous in terms of molecular aberrations, and oncogenesis could originate from chromosomal CNV and manifest as transcriptional alterations. However, relationships between DNA structural variations and mRNA abundance are not always linear, and complex regulatory mechanisms have rarely been addressed, further highlighting the necessity of identifying genes underpinning CRC tumorigenesis. GE studies using microarray, RT–PCR, or digital RNA counting have been advocated and performed widely to search biomarkers for cancer prognostic prediction [2–11, 17, 18]. Fresh frozen samples of newly diagnosed CRC patients were assayed in an integrated approach to synthesize the purposed concurrent gene signature, which could be used to improve CRC risk stratification, further augmenting treatment outcomes.

The 32 Taiwanese CRC patients assayed with SNP microarrays showed that the most frequent CNVs by GISTIC were 13q12.2-q12.3, and 17q12-q21.2. SNP array-based CNV analysis, which is a molecular cytogenetic method, can detect abnormalities in the number of copies of segments of tumor DNA, with losses or gains from assayed samples indicated from spots showing aberrant intensity signal ratios. Whole-genome SNP arrays can provide insight into the fundamental process of chromosomal instability leading to CRC oncogenesis [30].

Chromosomal aberrations seem to play a major role in regulating transcription [31, 32]. Since genomic imbalance would have a substantial impact on GE, the interplay between CNV and certain GE patterns for sporadic CRC might shed light on underlying molecular processes and the discovery of cancer-related prognostic genes. The main hypothesis underpinning the concurrent gene signature is that cancer may be bred at the chromosomal level with CNV and modulate subsequent GE profiles. Concurrent genes, which were designated to those displaying coherent patterns between tumor genomic and transcriptional alterations, were the filtered candidates for prognostic signature synthesis.

With the prevalence of high-throughput GE studies, hundreds of thousands of genes were measured in a single experiment, and gene filtering became inevitable to derive a clinically applicable signature from high-dimensional GE data [33]. In the current study, concurrent genes were the selection criteria for biomarker discovery and classifier development to identify potential candidates through algorithms integrating SNP and GE microarrays. Both CNV and GE data were available in 31 subjects with Spearman’s correlation coefficients calculated for each gene, resulting in 1582 concurrent genes (*P* < 0.01). The secondary discovery cohort, comprising 345 patients from TCGA-COAD project, revealed 2974 concurrent genes using the same algorithm (*P* < 0.01). Instead of using the 307 intersecting genes, we combined correlations from two independent cohorts using z-transformation, and 4022 genes were filtered (*P* < 0.001). Calling pipelines were the same for both discovery cohorts once the GE abundance and CNV were summarized from a gene-by gene basis, despite different microarray platforms were adopted within each study. The z-transformed correlation coefficients were used to identify the universal concurrent genes in an effort to overcome the unbalanced sample size between two discovery cohorts. This sophisticated statistical framework addressed the sample size discrepancy between two discovery cohorts without losing generalizability.

Publicly available CRC microarray studies were gathered from the NCBI’s Gene Expression Omnibus. Studies reporting relapse-free survival or overall survival, as well as those with dichotomous prognostic outcomes, were included. Processed data were downloaded and analyzed without further modification except for gene centering, since prognostic comparisons were performed within each dataset to avoid batch effects across microarray studies. The 4022 candidate concurrent genes were evaluated within each of the 10 microarray datasets, and 1655 (2000 probe sets) were recognized as being prognostic in at least one study. The final prognostic model was the consensus across 10 microarray datasets. Distinct relapse-free or overall survival patterns were evident from four out of five datasets, and the predictive accuracy of adverse events was between 61 and 86% from another five independent studies. It deserves notice that GE-based predicted risk is always continuous, that’s why an arbitrary threshold for dichotomous stratification is needed for prognostication. The 50th percentile was selected as an uninformative prior, which meant that there was equal chance of being censored/event during survival analysis. With high variability of censored cases across studies (68% ~ 92%), our sensitivity analyses showed that an uninformative prior set to the 50th percentile of the prognostic index score might be a better choice to enhance generalizability for real-world practice. The built-in multiple-methods of the BRB-ArrayTools were adopted for class prediction. For high-dimensional GE data, there is no gold standard for which is the best method for class prediction, so we conducted exhaustive bioinformatics approaches to identify the best model.

As pointed out by Marshall et al. from a review article regarding multi-omics, after 10 years’ progress of tumor mutational and transcriptional profiling in CRC, the prognostic power of modern genetic testing brings only modest benefits in terms of treatment guidance, i.e., who will benefit from adjuvant chemotherapy and if so, what is the optimal duration or intensity? [34]. Indeed, there remains an unmet need of precise risk estimation for proper management of CRC patients while our study provided a plausible multi-gene expression signature for such task. Survival rate of stage II CRC is around 60 to 85% while 25% of patients of this stage will relapse, and once relapse happened, their survival rate will drop drastically. Consequently, guiding the decision of adjuvant cytotoxic chemotherapy for CRC is one of the major priorities for the application of multi-gene expression-based testing.

Therefore, we developed the prognostic model for CRC as an initiative toward personalized and precision medicine.

The biological relevance of the 49 constitutional genes was deciphered. *KDM6B* (*JMJD3*) is an epigenetic gene coding for a histone demethylase and is also a VDR co-target that partially mediates the effects of 1,25-(OH)_2_D_3_ on the human colon [35]. *ATAD5* mediates the cellular response to DNA damage [36]. *HIP1R* has been harvested from sera of CRC but not from normal blood donors [37]. *PBK* is a serine/threonine kinase, and its expression is elevated in breast cancer, prostate cancer, and CRC [38]. PBK/TOPK interacts with the DBD domain of the tumor suppressor p53 and modulates the expression of transcriptional targets, including p21 [39]. *PIAS2* regulates the IFN-gamma signaling pathway, affecting tumor development in non-small-cell lung cancer [40]. *FLNA*, *DUSP14*, and *FAS* are implicated in the MAPK pathway [41]. *FAS* is relevant to p53 and the apoptosis pathway [42], and *THBS1* interacts with TGF-beta in glioblastoma [43]. *POLD2* is involved in DNA replication and mismatch/base excision repair [44]. Finally, *CSTF1* participates in mRNA polyadenylation [45]. In addition, many signature genes remained undetermined regarding their roles in CRC pathogenesis, while our integrated analysis was an initial step toward understanding their relevance in CRC initiation and progression.

There were some limitations of the study. First, as sequencing was not conducted, it was not possible to evaluate the impact of tumor DNA sequence variants upon GE; consequently, genes impacted by both mutations and CNV were not selected for signature construction. Future prospective validation studies using retrospective formalin-fixed paraffin-embedded (FFPE) samples are warranted to show the true prognostic value of concurrent gene signatures. The benefits of FFPE samples include readily available pathological archives, affordable quantitative RT–PCR testing or digital RNA counting rather than much more expensive microarrays, and an abundance of clinical information from retrospective cohorts. We hope the integrated approach could lead to the discovery of potential biomarkers with prognostic value for CRC to determine the most efficient adjuvant therapy based on risk stratification, especially for stage II patients [46]. With prognostic validation of the concurrent gene signature, those predicted into the high-risk group should be managed with postoperative adjuvant therapy to reduce their risk of recurrence and metastasis and should be assayed by targeted sequencing of actionable mutations such as *KRAS* and *BRAF* [47]. On the other hand, stage II CRC patients categorized into the low-risk group by concurrent gene signature may avoid toxic chemotherapy under regular postoperative surveillance.

## Conclusions

The concurrent gene risk predictive model is prognostic for CRC recurrence, metastasis, or mortality as well as relapse-free/overall survival from 1746 patients. CRC oncogenesis might originate from tumor CNV and manifest through transcription as GE profiles. Genes with coherent patterns between chromosomal and transcriptional contexts are more likely to serve as potential biomarkers for sporadic CRC. With prognostic validation of the concurrent gene signature, more precise risk assessment will be achieved to overcome the molecular heterogeneity of CRC, and the results will provide further opportunities for personalized therapy.

## Supplementary Information

**Additional file 1: Supplementary Table 1.** Microarray datasets with survival outcomes (*n* = 1331). **Supplementary Table 2.** Microarray datasets with dichotomous outcomes pertaining to adverse events (*n* = 415). **Supplementary Table 3.** Pathways enrichment in genes with gain and loss. **Supplementary Table 4.** Gain and loss regions and associated genes by GISTIC among 32 Taiwanese CRC patients (GISTIC: Genomic Identification of Significant Targets in Cancer, CRC: colorectal cancer). **Supplementary Table 5.** Complete list of the concurrent gene signature. **Supplementary Table 6.** Summary of censored case numbers in high- and low-risk groups and area under the curve (AUC) from time-dependent receiver operating characteristic (ROC) curve from survival analysis (gene only model, RFS: relapse-free survival, OS: Overall survival, CRC: colorectal cancer, GEO: gene expression omnibus) with risk-group thresholding set to the 75th percentile.

**Additional file 2: Supplementary Figure 1.** Gain-loss plot of the genome of 32 Taiwanese CRC SNP microarrays (CRC: colorectal cancer SNP: single nucleotide polymorphism). **Supplemental Figure 2.** A-E Relapse-free/overall survival analysis from microarray datasets of GSE12945 (2A, top left), GSE14333 (2B, top right), GSE17538 (2C, middle left), TCGA_COAD (2D, middle right), and GSE39582 (2E, bottom) with leave-one-out cross-validation. The high−/low-risk group was defined by the 75th percentile of the prognostic index score determined by the supervised principal component composed of 49 concurrent genes. All survival times were measured in months, except for 3D, which was measured in days.

**Additional file 3.**

## Data Availability

Processed gene expression values and a gene-centric table pertaining the average log-intensity ratio per gene from comparative genomic hybridization experiments (SNP microarrays), as well as sample information are detailed in the Additional file 3. Raw microarray data of Taiwanese CRC is deposited to the NCBI GEO with the accession number GSE197802.

## Abbreviations

SNP: Single nucleotide polymorphism
GE: Gene expression
CNV: Copy number variation
TCGA: The Cancer Genome Atlas project
ROC: Receiver operating characteristic
CRC: Colorectal cancer
RT–PCR: Reserve transcription-polymerase chain reaction
FAP: Familial adenomatous polyposis
IRB: Institutional review board
MAF: Minor allele frequency
CBS: Circular binary segmentation
MAD: Median absolute deviation
GISTIC: Genomic identification of significant targets in cancer
COAD: Colon adenocarcinoma
SVM: Support vector machine
FFPE: Formalin-fixed paraffin-embedded
