# Supplementary material for: Colorectal cancer concurrent gene signature based on coherent patterns between genomic and transcriptional alterations
Source: BMC Cancer. 2022 May 30;22:590. doi: 10.1186/s12885-022-09627-9 (PMC9150289; doi:10.1186/s12885-022-09627-9)
Supplement: Supplementary file 1 — Additional file 1: Supplementary Table 1. Microarray datasets with survival outcomes (n = 1331). Supplementary Table 2. Microarray datasets with dichotomous outcomes pertaining to adverse events (n = 415). Supplementary Table 3. Pathways enrichment in genes with gain and loss. Supplementary Table 4. Gain and loss regions and associated genes by GISTIC among 32 Taiwanese CRC patients (GISTIC: Genomic Identification of Significant Targets in Cancer, CRC: colorectal cancer). Supplementary Table 5. Complete list of the concurrent gene signature. Supplementary Table 6. Summary of censored case numbers in high- and low-risk groups and area under the curve (AUC) from time-dependent receiver operating characteristic (ROC) curve from survival analysis (gene only model, RFS: relapse-free survival, OS: Overall survival, CRC: colorectal cancer, GEO: gene expression omnibus) with risk-group thresholding set to the 75th percentile. [file 12885_2022_9627_MOESM1_ESM.docx]

Supplementary Files

Additional File 1. Supplementary Tables 1-6.

Supplementary Table 1. Microarray datasets with survival outcomes (n=1331).

| **GEO/dataset** | 12945 | 14333 | 17538 | 39582 | TCGA_COAD |
| --- | --- | --- | --- | --- | --- |
|  |  |  |  |  |  |
| **Sample size** | 62 | 290 | 238(232*) | 566 | 181 |
|  |  |  |  |  |  |
| **Outcomes** | OS/RFS | RFS | OS/RFS | OS/RFS | OS |
|  |  |  |  |  |  |
| **Features** | Pathological stage, grade, nodal status | Duke stage | Stage | Stage, MMR, P53,  Kras, BARF status | Stage, LVI, Kras,  BRAF, ALK, EGFR, MSI status |
|  |  |  |  |  |  |
| **Array platform** | HG-U133A | HG-U133+ 2.0 | HG-U133+ 2.0 | HG-U133+ 2.0 | Agilent G4502A |

*Samples without missing values

Supplementary Table 2. Microarray datasets with dichotomous outcomes pertaining to adverse events (n=415).

| **GEO/dataset** | 5206 | 9348 | 18088 | 18105 | 64857 |
| --- | --- | --- | --- | --- | --- |
|  |  |  |  |  |  |
| **Sample size** | 105(100*) | 82(70*) | 53 | 111 | 81 |
|  |  |  |  |  |  |
| **Outcomes** | Recurrence | Metastasis | Recurrence | Metastasis | Recurrence |
|  |  |  |  |  |  |
| **Features** | Stage, Duke stage, grade |  | Grade, MSI, Stage II | Stage II+III |  |
|  |  |  |  |  |  |
| **Array platform** | HG-U133+ 2.0 | HG-U133+ 2.0 | HG-U133+ 2.0 | HG-U133+ 2.0 | HG-U133+ 2.0 |

*Samples without missing values

Supplementary Table 3. Pathways enrichment in genes with gain and loss.

Pathway enrichment in genes with gain:

|  | Pathway | Description | Number of genes | Gene symbol | P-value |
| --- | --- | --- | --- | --- | --- |
| 1 | h_myosinPathway | [PKC-catalyzed phosphorylation of inhibitory phosphoprotein of myosin phosphatase](http://cgap.nci.nih.gov/Pathways/BioCarta/h_myosinPathway) | 11 | [*PPP1R12B*](http://www.ncbi.nlm.nih.gov/entrez/query.fcgi?cmd=search&db=gene&term=PPP1R12B)*,* [*GNA12*](http://www.ncbi.nlm.nih.gov/entrez/query.fcgi?cmd=search&db=gene&term=GNA12)*,* [*GNAQ*](http://www.ncbi.nlm.nih.gov/entrez/query.fcgi?cmd=search&db=gene&term=GNAQ)*,* [*ARHGAP5*](http://www.ncbi.nlm.nih.gov/entrez/query.fcgi?cmd=search&db=gene&term=ARHGAP5)*,* [*PRKCB*](http://www.ncbi.nlm.nih.gov/entrez/query.fcgi?cmd=search&db=gene&term=PRKCB)*,* [*GNA13*](http://www.ncbi.nlm.nih.gov/entrez/query.fcgi?cmd=search&db=gene&term=GNA13)*,* [*PRKCA*](http://www.ncbi.nlm.nih.gov/entrez/query.fcgi?cmd=search&db=gene&term=PRKCA)*,* [*ROCK1*](http://www.ncbi.nlm.nih.gov/entrez/query.fcgi?cmd=search&db=gene&term=ROCK1)*,* [*PKN1*](http://www.ncbi.nlm.nih.gov/entrez/query.fcgi?cmd=search&db=gene&term=PKN1)*,* [*ARHGEF1*](http://www.ncbi.nlm.nih.gov/entrez/query.fcgi?cmd=search&db=gene&term=ARHGEF1)*,* [*PLCB1*](http://www.ncbi.nlm.nih.gov/entrez/query.fcgi?cmd=search&db=gene&term=PLCB1) | 0.007 |

Pathway enrichment in genes with loss:

|  | Pathway | Description | Number of genes | Gene symbol | P-value |
| --- | --- | --- | --- | --- | --- |
| 1 | h_skp2e2fPathway | [E2F1 Destruction Pathway](http://cgap.nci.nih.gov/Pathways/BioCarta/h_skp2e2fPathway) | 10 | [*SKP2*](http://www.ncbi.nlm.nih.gov/entrez/query.fcgi?cmd=search&db=gene&term=SKP2)*,* [*SKP1*](http://www.ncbi.nlm.nih.gov/entrez/query.fcgi?cmd=search&db=gene&term=SKP1)*,* [*CUL1*](http://www.ncbi.nlm.nih.gov/entrez/query.fcgi?cmd=search&db=gene&term=CUL1)*,* [*CDK2*](http://www.ncbi.nlm.nih.gov/entrez/query.fcgi?cmd=search&db=gene&term=CDK2)*,* [*CCNA1*](http://www.ncbi.nlm.nih.gov/entrez/query.fcgi?cmd=search&db=gene&term=CCNA1)*,* [*RB1*](http://www.ncbi.nlm.nih.gov/entrez/query.fcgi?cmd=search&db=gene&term=RB1)*,* [*TFDP1*](http://www.ncbi.nlm.nih.gov/entrez/query.fcgi?cmd=search&db=gene&term=TFDP1)*,* [*CDC34*](http://www.ncbi.nlm.nih.gov/entrez/query.fcgi?cmd=search&db=gene&term=CDC34)*,* [*CCNE1*](http://www.ncbi.nlm.nih.gov/entrez/query.fcgi?cmd=search&db=gene&term=CCNE1)*,* [*E2F1*](http://www.ncbi.nlm.nih.gov/entrez/query.fcgi?cmd=search&db=gene&term=E2F1) | 0.002 |
| 2 | h_fbw7Pathway | [Cyclin E Destruction Pathway](http://cgap.nci.nih.gov/Pathways/BioCarta/h_fbw7Pathway) | 9 | [*FBXW7*](http://www.ncbi.nlm.nih.gov/entrez/query.fcgi?cmd=search&db=gene&term=FBXW7)*,* [*SKP1*](http://www.ncbi.nlm.nih.gov/entrez/query.fcgi?cmd=search&db=gene&term=SKP1)*,* [*CUL1*](http://www.ncbi.nlm.nih.gov/entrez/query.fcgi?cmd=search&db=gene&term=CUL1)*,* [*CDK2*](http://www.ncbi.nlm.nih.gov/entrez/query.fcgi?cmd=search&db=gene&term=CDK2)*,* [*RB1*](http://www.ncbi.nlm.nih.gov/entrez/query.fcgi?cmd=search&db=gene&term=RB1)*,* [*TFDP1*](http://www.ncbi.nlm.nih.gov/entrez/query.fcgi?cmd=search&db=gene&term=TFDP1)*,* [*CDC34*](http://www.ncbi.nlm.nih.gov/entrez/query.fcgi?cmd=search&db=gene&term=CDC34)*,* [*CCNE1*](http://www.ncbi.nlm.nih.gov/entrez/query.fcgi?cmd=search&db=gene&term=CCNE1)*,* [*E2F1*](http://www.ncbi.nlm.nih.gov/entrez/query.fcgi?cmd=search&db=gene&term=E2F1) | 0.004 |

Supplementary Table 4. Gain and loss regions and associated genes by GISTIC among 32 Taiwanese CRC patients (GISTIC: Genomic Identification of Significant Targets in Cancer, CRC: colorectal cancer).

Table of gain regions:

|  | Chromosome | Start | End | Cytoband | Number of genes | Gene symbol |
| --- | --- | --- | --- | --- | --- | --- |
| 1 | 7 | 61985092 | 61989559 | 7q11.21 | 0 |  |
| 2 | 13 | 27976955 | 27985200 | 13q12.2 | 0 |  |
| 3 | 13 | 28411055 | 29120052 | 13q12.2-q12.3 | 8 | [*PDX1*](http://www.ncbi.nlm.nih.gov/entrez/query.fcgi?cmd=search&db=gene&term=PDX1)*,* [*ATP5EP2*](http://www.ncbi.nlm.nih.gov/entrez/query.fcgi?cmd=search&db=gene&term=ATP5EP2)*,* [*CDX2*](http://www.ncbi.nlm.nih.gov/entrez/query.fcgi?cmd=search&db=gene&term=CDX2)*,* [*PRHOXNB*](http://www.ncbi.nlm.nih.gov/entrez/query.fcgi?cmd=search&db=gene&term=PRHOXNB)*,* [*FLT3*](http://www.ncbi.nlm.nih.gov/entrez/query.fcgi?cmd=search&db=gene&term=FLT3)*,* [*LOC100288730*](http://www.ncbi.nlm.nih.gov/entrez/query.fcgi?cmd=search&db=gene&term=LOC100288730)*,* [*PAN3*](http://www.ncbi.nlm.nih.gov/entrez/query.fcgi?cmd=search&db=gene&term=PAN3)*,* [*FLT1*](http://www.ncbi.nlm.nih.gov/entrez/query.fcgi?cmd=search&db=gene&term=FLT1) |
| 4 | 13 | 37422417 | 37422515 | 13q13.3 | 1 | [*SMAD9*](http://www.ncbi.nlm.nih.gov/entrez/query.fcgi?cmd=search&db=gene&term=SMAD9) |
| 5 | 14 | 106787376 | 106795391 | 14q32.33 | 0 |  |
| 6 | 15 | 25924539 | 25924798 | 15q12 | 1 | [*ATP10A*](http://www.ncbi.nlm.nih.gov/entrez/query.fcgi?cmd=search&db=gene&term=ATP10A) |
| 7 | 16 | 28618318 | 28620752 | 16p11.2 | 1 | [*SULT1A1*](http://www.ncbi.nlm.nih.gov/entrez/query.fcgi?cmd=search&db=gene&term=SULT1A1) |
| 8 | 16 | 70723353 | 70723380 | 16q22.1 | 1 | [*VAC14*](http://www.ncbi.nlm.nih.gov/entrez/query.fcgi?cmd=search&db=gene&term=VAC14) |
| 9 | 17 | 37711565 | 38114221 | 17q12-q21.1 | 14 | [*NEUROD2*](http://www.ncbi.nlm.nih.gov/entrez/query.fcgi?cmd=search&db=gene&term=NEUROD2)*,* [*PPP1R1B*](http://www.ncbi.nlm.nih.gov/entrez/query.fcgi?cmd=search&db=gene&term=PPP1R1B)*,* [*STARD3*](http://www.ncbi.nlm.nih.gov/entrez/query.fcgi?cmd=search&db=gene&term=STARD3)*,* [*TCAP*](http://www.ncbi.nlm.nih.gov/entrez/query.fcgi?cmd=search&db=gene&term=TCAP)*,* [*PNMT*](http://www.ncbi.nlm.nih.gov/entrez/query.fcgi?cmd=search&db=gene&term=PNMT)*,* [*PGAP3*](http://www.ncbi.nlm.nih.gov/entrez/query.fcgi?cmd=search&db=gene&term=PGAP3)*,* [*ERBB2*](http://www.ncbi.nlm.nih.gov/entrez/query.fcgi?cmd=search&db=gene&term=ERBB2)*,* [*C17orf37*](http://www.ncbi.nlm.nih.gov/entrez/query.fcgi?cmd=search&db=gene&term=C17orf37)*,* [*GRB7*](http://www.ncbi.nlm.nih.gov/entrez/query.fcgi?cmd=search&db=gene&term=GRB7)*,* [*IKZF3*](http://www.ncbi.nlm.nih.gov/entrez/query.fcgi?cmd=search&db=gene&term=IKZF3)*,* [*ZPBP2*](http://www.ncbi.nlm.nih.gov/entrez/query.fcgi?cmd=search&db=gene&term=ZPBP2)*,* [*GSDMB*](http://www.ncbi.nlm.nih.gov/entrez/query.fcgi?cmd=search&db=gene&term=GSDMB)*,* [*ORMDL3*](http://www.ncbi.nlm.nih.gov/entrez/query.fcgi?cmd=search&db=gene&term=ORMDL3)*,* [*LOC728129*](http://www.ncbi.nlm.nih.gov/entrez/query.fcgi?cmd=search&db=gene&term=LOC728129) |
| 10 | 17 | 38136026 | 38174209 | 17q21.1 | 2 | [*PSMD3*](http://www.ncbi.nlm.nih.gov/entrez/query.fcgi?cmd=search&db=gene&term=PSMD3)*,* [*CSF3*](http://www.ncbi.nlm.nih.gov/entrez/query.fcgi?cmd=search&db=gene&term=CSF3) |
| 11 | 17 | 38616358 | 38638251 | 17q21.2 | 1 | [*TNS4*](http://www.ncbi.nlm.nih.gov/entrez/query.fcgi?cmd=search&db=gene&term=TNS4) |
| 12 | 17 | 38648405 | 38689032 | 17q21.2 | 1 | [*TNS4*](http://www.ncbi.nlm.nih.gov/entrez/query.fcgi?cmd=search&db=gene&term=TNS4) |
| 13 | 20 | 36844881 | 36845715 | 20q11.23 | 1 | [*KIAA1755*](http://www.ncbi.nlm.nih.gov/entrez/query.fcgi?cmd=search&db=gene&term=KIAA1755) |
| 14 | 20 | 58089857 | 58090667 | 20q13.32 | 0 |  |

Table of loss regions:

|  | Chromosome | Start | End | Cytoband | Number of genes | Gene symbol |
| --- | --- | --- | --- | --- | --- | --- |
| 1 | 1 | 12858008 | 12870465 | 1p36.21 | 0 |  |
| 2 | 1 | 95524503 | 95524551 | 1p21.3 | 1 | [*ALG14*](http://www.ncbi.nlm.nih.gov/entrez/query.fcgi?cmd=search&db=gene&term=ALG14) |
| 3 | 1 | 245442313 | 245443262 | 1q44 | 1 | [*KIF26B*](http://www.ncbi.nlm.nih.gov/entrez/query.fcgi?cmd=search&db=gene&term=KIF26B) |
| 4 | 3 | 56608033 | 56616795 | 3p14.3 | 1 | [*CCDC66*](http://www.ncbi.nlm.nih.gov/entrez/query.fcgi?cmd=search&db=gene&term=CCDC66) |
| 5 | 3 | 148964692 | 148969220 | 3q25.1 | 0 |  |
| 6 | 4 | 31464058 | 31466197 | 4p15.1 | 0 |  |
| 7 | 4 | 58256666 | 58261728 | 4q12 | 0 |  |
| 8 | 4 | 98175392 | 98186340 | 4q22.3 | 0 |  |
| 9 | 4 | 161014288 | 161019327 | 4q32.1 | 0 |  |
| 10 | 4 | 182969157 | 182970006 | 4q34.3 | 0 |  |
| 11 | 5 | 70305696 | 70307386 | 5q13.2 | 1 | [*NAIP*](http://www.ncbi.nlm.nih.gov/entrez/query.fcgi?cmd=search&db=gene&term=NAIP) |
| 12 | 5 | 146394824 | 146397165 | 5q32 | 1 | [*PPP2R2B*](http://www.ncbi.nlm.nih.gov/entrez/query.fcgi?cmd=search&db=gene&term=PPP2R2B) |
| 13 | 5 | 178387820 | 178390407 | 5q35.3 | 1 | [*ZNF454*](http://www.ncbi.nlm.nih.gov/entrez/query.fcgi?cmd=search&db=gene&term=ZNF454) |
| 14 | 6 | 3621539 | 3621551 | 6p25.2 | 0 |  |
| 15 | 6 | 77016855 | 77024665 | 6q14.1 | 0 |  |
| 16 | 6 | 98597689 | 98597809 | 6q16.1 | 0 |  |
| 17 | 6 | 118077577 | 118084347 | 6q22.1 | 0 |  |
| 18 | 6 | 154826291 | 154826460 | 6q25.2 | 1 | [*CNKSR3*](http://www.ncbi.nlm.nih.gov/entrez/query.fcgi?cmd=search&db=gene&term=CNKSR3) |
| 19 | 7 | 62688981 | 62729224 | 7q11.21 | 0 |  |
| 20 | 7 | 86238045 | 86244535 | 7q21.11 | 0 |  |
| 21 | 8 | 32153164 | 32153473 | 8p12 | 1 | [*NRG1*](http://www.ncbi.nlm.nih.gov/entrez/query.fcgi?cmd=search&db=gene&term=NRG1) |
| 22 | 9 | 83071931 | 83071954 | 9q21.31 | 0 |  |
| 23 | 10 | 90941441 | 90945998 | 10q23.31 | 0 |  |
| 24 | 12 | 69557876 | 69558981 | 12q15 | 0 |  |
| 25 | 12 | 132987231 | 132987463 | 12q24.33 | 0 |  |
| 26 | 14 | 44504048 | 44531417 | 14q21.2 | 0 |  |
| 27 | 15 | 23090818 | 23109890 | 15q11.2 | 0 |  |
| 28 | 15 | 63267778 | 63268686 | 15q22.2 | 0 |  |
| 29 | 16 | 3700815 | 3708193 | 16p13.3 | 2 | [*DNASE1*](http://www.ncbi.nlm.nih.gov/entrez/query.fcgi?cmd=search&db=gene&term=DNASE1)*,* [*TRAP1*](http://www.ncbi.nlm.nih.gov/entrez/query.fcgi?cmd=search&db=gene&term=TRAP1) |
| 30 | 16 | 85997028 | 85997281 | 16q24.1 | 0 |  |
| 31 | 17 | 6996653 | 7002483 | 17p13.1 | 0 |  |
| 32 | 18 | 21078716 | 21079939 | 18q11.2 | 0 |  |
| 33 | 18 | 64777373 | 64777529 | 18q22.1 | 0 |  |
| 34 | 18 | 66747568 | 66755736 | 18q22.1 | 0 |  |
| 35 | 18 | 76528007 | 76528068 | 18q23 | 0 |  |

Supplementary Table 5. Complete list of the concurrent gene signature.

| Gene symbol | Gene name | EntrezID | Accession | UGCluster | Defined gene list |
| --- | --- | --- | --- | --- | --- |
| NDUFA8 | NADH dehydrogenase (ubiquinone) 1 alpha subcomplex, 8, 19kDa | 4702 | NM_014222 | Hs.495039 | Alzheimer's disease, Huntington's disease, Metabolic pathways, Oxidative phosphorylation, Parkinson's disease |
| RAB31 | RAB31, member RAS oncogene family | 11031 | BE789881 | Hs.99528 | Endocytosis |
| FLNA | filamin A, alpha | 2316 | NM_001456 | Hs.195464 | Focal adhesion, MAPK signaling pathway |
| BRI3BP | BRI3 binding protein | 140707 | AI357639 | Hs.596464 |  |
| PTPN14 | protein tyrosine phosphatase, non-receptor type 14 | 5784 | AW129783 | Hs.193557 |  |
| ATAD5 | ATPase family, AAA domain containing 5 | 79915 | NM_024857 | Hs.528902 |  |
| DUSP14 | dual specificity phosphatase 14 | 11072 | NM_007026 | Hs.91448 | MAPK signaling pathway |
| VLDLR | very low density lipoprotein receptor | 7436 | L22431 | Hs.370422 | Reelin Signaling Pathway |
| DDC | dopa decarboxylase (aromatic L-amino acid decarboxylase) | 1644 | NM_000790 | Hs.359698 | Histidine metabolism, Metabolic pathways, Phenylalanine metabolism, Tryptophan metabolism, Tyrosine metabolism |
| CSTF1 | cleavage stimulation factor, 3' pre-RNA, subunit 1, 50kDa | 1477 | L02547 | Hs.172865 | Polyadenylation of mRNA, mRNA surveillance pathway |
| IDH3B | isocitrate dehydrogenase 3 (NAD+) beta | 3420 | NM_006899 | Hs.436405 | Citrate cycle (TCA cycle), Metabolic pathways |
| SALL1 | spalt-like transcription factor 1 | 6299 | AU152837 | Hs.135787 |  |
| POLD2 | polymerase (DNA directed), delta 2, accessory subunit | 5425 | NM_006230 | Hs.306791 | Base excision repair, DNA replication, Homologous recombination, Metabolic pathways, Mismatch repair, Nucleotide excision repair, Purine metabolism, Pyrimidine metabolism |
| CENPB | centromere protein B, 80kDa | 1059 | AL109804 | Hs.516855 |  |
| HIP1R | huntingtin interacting protein 1 related | 9026 | AB013384 | Hs.524815 |  |
| SLC25A15 | solute carrier family 25 (mitochondrial carrier; ornithine transporter) member 15 | 10166 | BC002702 | Hs.646645 |  |
| CDCA2 | cell division cycle associated 2 | 157313 | T90295 | Hs.33366 |  |
| MOCS3 | molybdenum cofactor synthesis 3 | 27304 | NM_014484 | Hs.159410 | Sulfur relay system |
| PSMB4 | proteasome (prosome, macropain) subunit, beta type, 4 | 5692 | AA630330 | Hs.89545 | Proteasome |
| DNAJC11 | DnaJ (Hsp40) homolog, subfamily C, member 11 | 55735 | BC014145 | Hs.462640 |  |
| FANCI | Fanconi anemia, complementation group I | 55215 | BG403615 | Hs.513126 |  |
| THBS1 | thrombospondin 1 | 7057 | AI812030 | Hs.164226 | TSP-1 Induced Apoptosis in Microvascular Endothelial Cell , Bladder cancer, ECM-receptor interaction, Focal adhesion, Malaria, p53 signaling pathway, Phagosome, TGF-beta signaling pathway |
| PBK | PDZ binding kinase | 55872 | NM_018492 | Hs.104741 |  |
| PDIK1L | PDLIM1 interacting kinase 1 like | 149420 | AI806633 | Hs.468801 |  |
| BAG6 | BCL2-associated athanogene 6 | 7917 | AW104426 | Hs.440900 |  |
| TCHP | trichoplein, keratin filament binding | 84260 | BC004285 | Hs.410924 |  |
| PMEPA1 | prostate transmembrane protein, androgen induced 1 | 56937 | AL035541 | Hs.517155 |  |
| PIAS2 | protein inhibitor of activated STAT, 2 | 9063 | AF361054 | Hs.657844 | Sumoylation by RanBP2 Regulates Transcriptional Repression, Hepatitis C, Jak-STAT signaling pathway, Pathways in cancer, Small cell lung cancer, Ubiquitin mediated proteolysis |
| METTL17 | methyltransferase like 17 | 64745 | AF321002 | Hs.512693 |  |
| GFM2 | G elongation factor, mitochondrial 2 | 84340 | AK025314 | Hs.277154 |  |
| COX5B | cytochrome c oxidase subunit Vb | 1329 | AI557312 | Hs.1342 | Alzheimer's disease, Cardiac muscle contraction, Huntington's disease, Metabolic pathways, Oxidative phosphorylation, Parkinson's disease |
| COX11 | COX11 cytochrome c oxidase copper chaperone | 1353 | AI376724 | Hs.591171 | Metabolic pathways, Oxidative phosphorylation |
| RPRD1A | regulation of nuclear pre-mRNA domain containing 1A | 55197 | T79568 | Hs.464912 |  |
| ISCA1 | iron-sulfur cluster assembly 1 | 81689 | NM_030940 | Hs.449291 |  |
| MFF | mitochondrial fission factor | 56947 | AF258660 | Hs.471528 |  |
| TLK1 | tousled-like kinase 1 | 9874 | AF116684 | Hs.744917 |  |
| ATP5C1 | ATP synthase, H+ transporting, mitochondrial F1 complex, gamma polypeptide 1 | 509 | BG232034 | Hs.271135 | Alzheimer's disease, Huntington's disease, Metabolic pathways, Oxidative phosphorylation, Parkinson's disease |
| ACAN | aggrecan | 176 | BC036445 | Hs.2159 |  |
| ZMYM6 | zinc finger, MYM-type 6 | 9204 | NM_145310 | Hs.533986 |  |
| FAS | Fas cell surface death receptor | 355 | X83493 | Hs.244139 | Antigen Dependent B Cell Activation, Bystander B Cell Activation, CTL mediated immune response against target cells , FAS signaling pathway ( CD95 ), HIV Induced T Cell Apoptosis, HIV-I Nef: negative effector of Fas and TNF, IL-2 Receptor Beta Chain in T cell Activation, Keratinocyte Differentiation, Regulation of transcriptional activity by PML, Stress Induction of HSP Regulation, African trypanosomiasis, Allograft rejection, Alzheimer's disease, Apoptosis, Autoimmune thyroid disease, Chagas disease (American trypanosomiasis), Cytokine-cytokine receptor interaction, Graft-versus-host disease, MAPK signaling pathway, Natural killer cell mediated cytotoxicity, p53 signaling pathway, Pathways in cancer, Type I diabetes mellitus |
| GRHPR | glyoxylate reductase/hydroxypyruvate reductase | 9380 | AK024386 | Hs.155742 | Glyoxylate and dicarboxylate metabolism, Metabolic pathways, Pyruvate metabolism |
| STAM2 | signal transducing adaptor molecule (SH3 domain and ITAM motif) 2 | 10254 | NM_005843 | Hs.17200 | Endocytosis, Jak-STAT signaling pathway |
| COX7C | cytochrome c oxidase subunit VIIc | 1350 | AA382702 | Hs.430075 | Alzheimer's disease, Cardiac muscle contraction, Huntington's disease, Metabolic pathways, Oxidative phosphorylation, Parkinson's disease |
| UBA6 | ubiquitin-like modifier activating enzyme 6 | 55236 | BC031637 | Hs.212774 | Ubiquitin mediated proteolysis |
| CEP70 | centrosomal protein 70kDa | 80321 | BC016050 | Hs.531962 |  |
| NDUFB2 | NADH dehydrogenase (ubiquinone) 1 beta subcomplex, 2, 8kDa | 4708 | AA699958 | Hs.655788 | Alzheimer's disease, Huntington's disease, Metabolic pathways, Oxidative phosphorylation, Parkinson's disease |
| PRR14 | proline rich 14 | 78994 | BE788667 | Hs.293629 |  |
| KDM6B | lysine (K)-specific demethylase 6B | 23135 | AI830331 | Hs.223678 |  |
| EHD2 | EH-domain containing 2 | 30846 | NM_014601 | Hs.744963 | Endocytosis |

Supplementary Table 6. Summary of censored case numbers in high- and low-risk groups and area under the curve (AUC) from time-dependent receiver operating characteristic (ROC) curve from survival analysis (gene only model, RFS: relapse-free survival, OS: Overall survival, CRC: colorectal cancer, GEO: gene expression omnibus) with risk-group thresholding set to the 75^th^ percentile.

| GEO | Predicted high-/low-risk patients | Censored cases | Censored in high-risk group | Censored in low-risk group | P-value  (Log-rank test) |
| --- | --- | --- | --- | --- | --- |
| GSE12945 | 11/40 | 47(92.2%) | 9(81.8%) | 38(95%) | 0.148 |
| GSE14333 | 55/171 | 176(77.9%) | 30(54.6%) | 146(85.4%) | <0.001 |
| GSE17538 | 55/150 | 145(72.5%) | 30(60%) | 115(76.7%) | 0.033 |
| TCGA_COAD | 46/128 | 158(90.8%) | 41(89.1%) | 117(91.4%) | 0.774 |
| GSE39582 | 139/418 | 380(68.2%) | 94(67.6%) | 286(68.4%) | 0.974 |
