# Supplementary material for: Colorectal cancer concurrent gene signature based on coherent patterns between genomic and transcriptional alterations
Source: BMC Cancer. 2022 May 30;22:590. doi: 10.1186/s12885-022-09627-9 (PMC9150289; doi:10.1186/s12885-022-09627-9)
Supplement: Supplementary file 2 — Additional file 2: Supplementary Figure 1. Gain-loss plot of the genome of 32 Taiwanese CRC SNP microarrays (CRC: colorectal cancer SNP: single nucleotide polymorphism). Supplemental Figure 2. A-E Relapse-free/overall survival analysis from microarray datasets of GSE12945 (2A, top left), GSE14333 (2B, top right), GSE17538 (2C, middle left), TCGA_COAD (2D, middle right), and GSE39582 (2E, bottom) with leave-one-out cross-validation. The high−/low-risk group was defined by the 75th percentile of the prognostic index score determined by the supervised principal component composed of 49 concurrent genes. All survival times were measured in months, except for 3D, which was measured in days. [file 12885_2022_9627_MOESM2_ESM.docx]

Supplementary File(s)

Additional File 2. Supplementary Figure 1 and Supplementary Figure 2


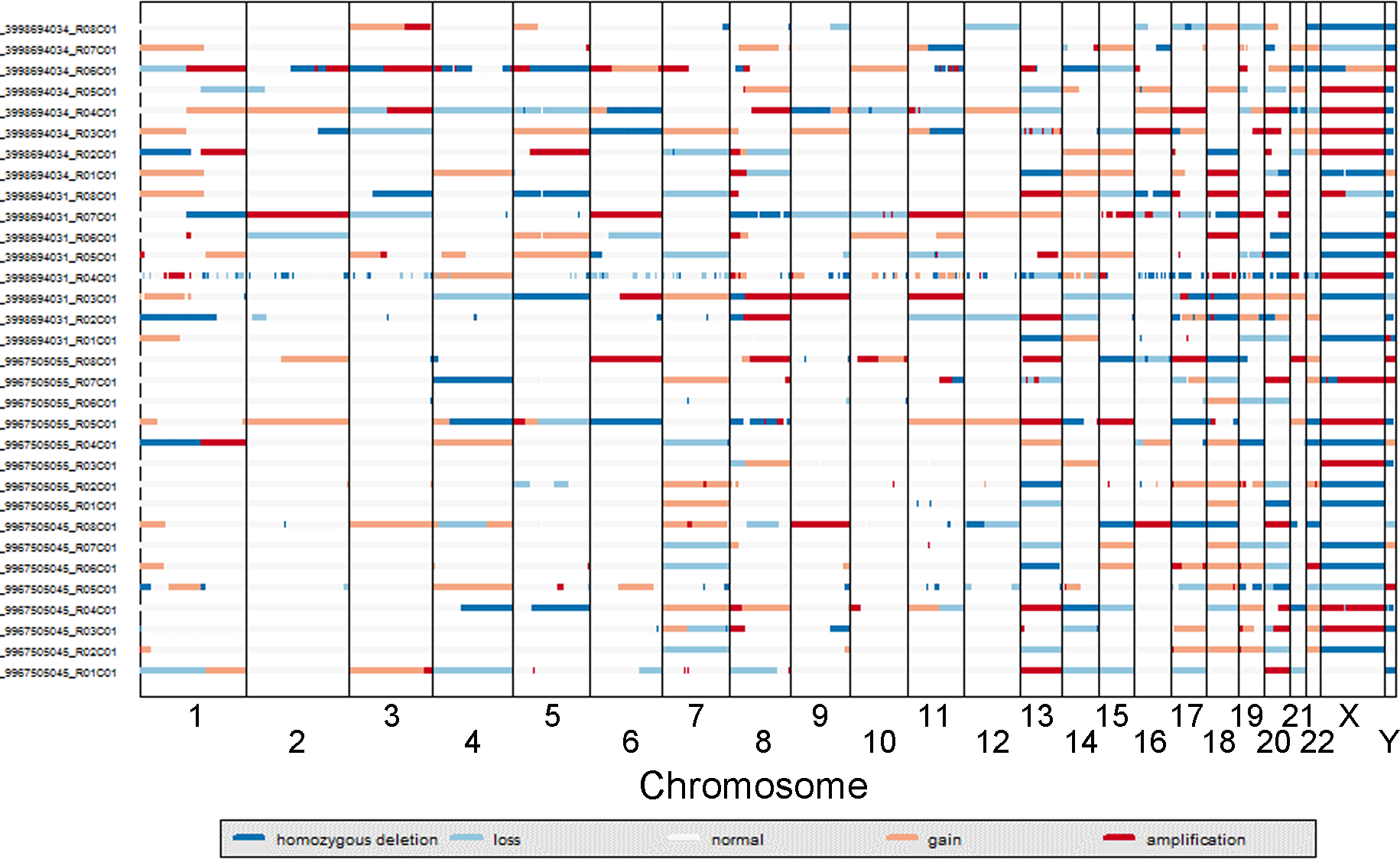


Supplementary Figure 1. Gain-loss plot of the genome of 32 Taiwanese CRC SNP microarrays (CRC: colorectal cancer SNP: single nucleotide polymorphism).


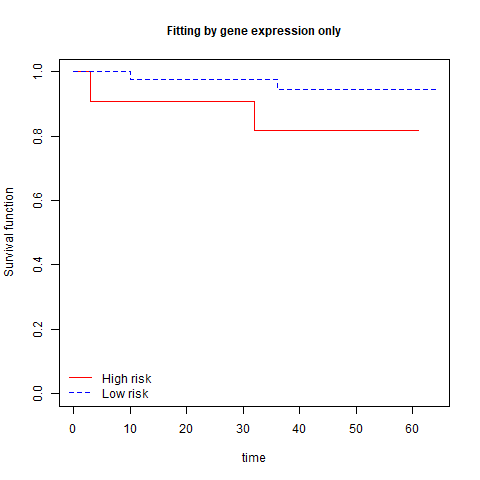

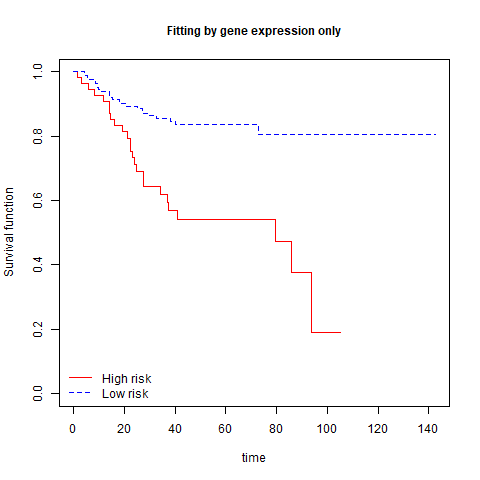


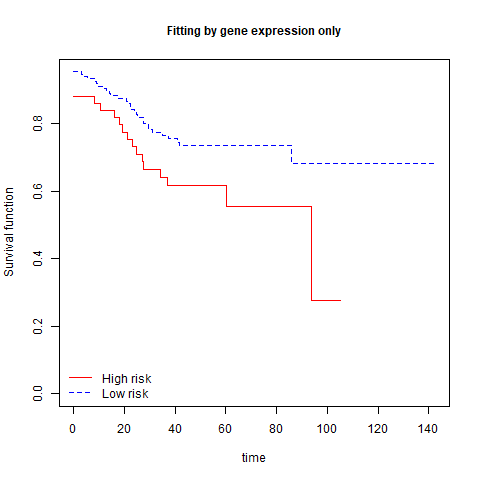

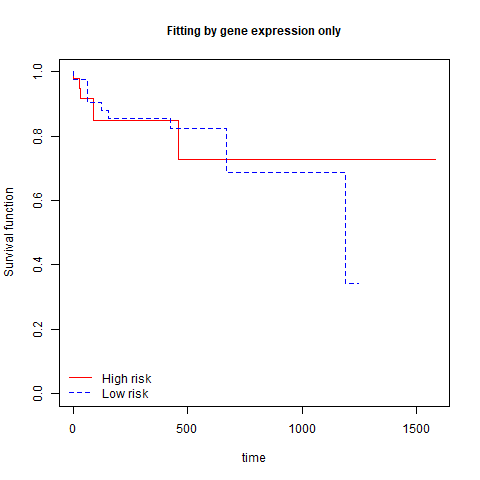


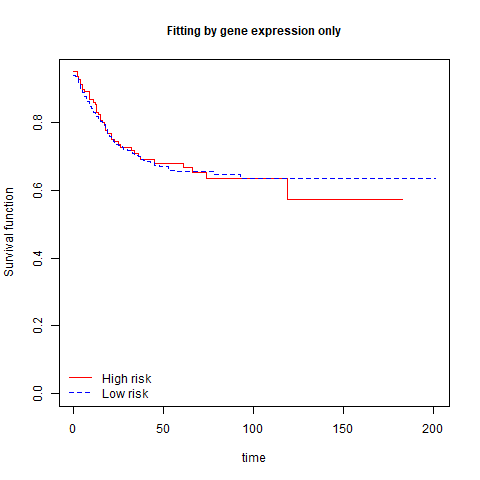


Supplemental Figure 2A to 2E. Relapse-free/overall survival analysis from microarray datasets of GSE12945 (2A, top left), GSE14333 (2B, top right), GSE17538 (2C, middle left), TCGA_COAD (2D, middle right), and GSE39582 (2E, bottom) with leave-one-out cross-validation. The high-/low-risk group was defined by the 75^th^ percentile of the prognostic index score determined by the supervised principal component composed of 49 concurrent genes. All survival times were measured in months, except for 3D, which was measured in days.
